# Young Women’s Silencing-Type Behaviors in Heterosexual Relationships

**DOI:** 10.1177/08862605241265417

**Published:** 2024-08-10

**Authors:** Tanja Samardzic, Paula C. Barata, Mavis Morton, Jeffery Yen

**Affiliations:** 1 University of Guelph, ON, Canada

**Keywords:** dating violence, domestic violence, youth violence, violence exposure

## Abstract

Feminist researchers have demonstrated that engagement in silencing of the self (i.e., self-restrictive and sacrificial behaviors reflecting how women “should” be in relationships) remains a prevalent strategy for relationship maintenance. However, little is known about (young) women silencing themselves in relationships where abuse is present. Young women’s experiences of silencing and other partner-focused behaviors (e.g., sexual compliance) within their relationships were thus explored. Young, partnered women (*M*_age_ = 21; *N* = 146) completed an online survey and open-ended questions about their current intimate relationships. Comparing between groups (abuse, *n* = 108; non-abuse, *n* = 38), the former scored higher on measures of total self-silencing, sexual compliance, and non-constructive communication and lower on measures of constructive communication (all *p* < .001). A mixed inductive and deductive content analysis found that while the importance of communicating with their partner was a preferred strategy for conflict management, multiple participants still emphasized self-suppression as an important part of their experience of relational conflict. Also, most participants indicated feeling as though they could be their authentic selves in their relationships, which suggests that their silencing may be situational and strategic in nature. These findings nuance previous understandings of self-silencing as inherently harmful and instead frame it as something sporadic and done strategically. They also bring forth questions about the extent to which young women’s emphasis on communication and insistence that they can be authentic are a product of changing societal expectations of women in today’s society compared to the 1980s/1990s when much of the foundational work on self-silencing was being done.

## An Understanding of the Self as Being in Relation

Since the late 20th century, feminist scholars have sought to understand women’s sense of themselves as being in relation to and increasingly focused on particularly intimate others (e.g., Gilligan, 1982; Jordan, 1997; Surrey, 1985, 1991). For instance, Miller (1976) discussed how young women’s sense of self becomes organized around their ability to develop and maintain (intimate) relationships. This suggests that a large part of young women’s sense of themselves and their self-worth is embedded within their ability to take care of intimate relationships, which is framed as a defining feature of being good (e.g., Gilligan, 2018; May, 2008).

Scholars have advanced research on women in relation by investigating how ideas of goodness are conflated with suppression and restriction in heterosexual relationships. For instance, Brown and Gilligan (1992) conducted foundational longitudinal work from 1986 to 1990 with nearly 100 girls aged 7 to 18 from the Lauren School for Girls in Cleveland, Ohio, of whom a sizable portion were girls of color and/or working class. Brown and Gilligan demonstrated that these girls took cues from important others in their lives and learned that being quiet was “good” behavior. Such early learning sets the stage for young women’s behaviors in intimate relationships and makes the achievement of healthy connections difficult. These findings reflected the riddle of femininity, where they were forced to choose between having a voice and maintaining important relationships (Gilligan, 2004). This paradoxical situation continues through adolescence and into adulthood (e.g., Samardzic et al., 2023).

## Self-Restriction in Context

The concept of self-silencing^1^ was constructed through Jack’s (1991) interviews with depressed women. Self-silencing has been conceived as the removal of critical aspects of oneself, such as one’s voice, for relational purposes. Women are said to internalize societal standards that equate selflessness with morality/goodness and femininity (e.g., Gilligan, 1982). Self-silencing is said to comprise a set of behaviors reflective of gender-specific schemas that guide women’s social behaviors and are calculated to achieve specific relational goals (Jack & Dill, 1992). Examples include over-caring for one’s partner and putting their needs above one’s own and active inhibition of self-expression for fear of conflict, retaliation, and/or loss of their intimate relationship (Impett & Peplau, 2002; Jack & Ali, 2010). Despite limited constructions of self-silencing as beneficial or strategic (e.g., beneficial for enhancing religiosity, spirituality, and relationship quality, Woodley, 2019; a strategy for resistance, Parpart & Parashar, 2019), there is general agreement that it is mostly harmful (e.g., Belenky et al., 1986; Jack, 1991). Its harm largely comes from an inability to express one’s wants, needs, feelings, etc., which greatly impacts relationship functioning via impaired communication and inauthenticity. When women silence themselves, they may even become more susceptible to diseases, such as eating disorders, and doing so may influence the resilience and therapeutic effects among patients with chronic conditions such as cancer (Maji & Dixit, 2018). Younger women are also disproportionately at risk for intimate partner violence (IPV) compared to older women (Smith et al., 2018). As such, young women’s silencing of themselves in the context of IPV may therefore impact their ability to experience authenticity in their relationships (Impett et al., 2008).

Some research has illuminated the association between self-silencing and IPV. Woods (1999) found that women’s physical and emotional abuse experiences were positively associated with self-silencing. Ali et al. (2000) found that emotional IPV was positively associated with self-silencing among women diagnosed with gastrointestinal concerns. Samardzic (2019) found that among young women students, self-silencing was positively associated with more frequent experiences of overall IPV. More recently, Inman and London (2022) observed a significant positive correlation between IPV victimization and self-silencing. Researchers have also demonstrated the impacts of self-silencing on other behaviors in relationships, like the increased likelihood of complying with unwanted sex (e.g., Widman et al., 2006) and poorer communication (e.g., Harper & Welsh, 2007). In Samardzic’s (2019) work, self-silencing was associated with constructive and partner demand/self-withdraw communication as well as sexual compliance among young women. Beyond these associations with IPV and a few other studies (e.g., Thompson et al., 2001; Whiffen et al., 2007), there is generally little known about young women’s silencing behaviors while in relationships with (abusive) men. What is specifically missing is a need to explore beyond the quantitative associations of self-silencing and other partner-focused behaviors by abuse experience, which in and of itself is important but lacks contextual information. Additional quantitative responses with qualitative information about specific situations in which young women engaged in such behaviors are needed to add this context.

## The Present Study

Given how central relationship maintenance is understood to be for young women (e.g., Samardzic et al., 2023), which is rooted in the enactment and performativity of gender and adherence to societal expectations, women may harness multiple strategies (e.g., sexual compliance and not speaking up) to maintain those relationships. In relationships where IPV may be present, these and other strategies (e.g., being careful about what to say to avoid their partner’s reaction; Goodman et al., 2003) may also be part of women’s experiences. But much of what is known of self-silencing in the context of IPV is correlational. Quantitative findings help illuminate how prevalent these behaviors are in women’s relationships and their associations with other related elements of experience, but qualitative data are needed to contextualize those behaviors.

We, thus, undertook a mixed methods approach and aimed to primarily analyze young women’s written descriptions about the times, in their intimate relationships, when they prioritized their partner’s needs and silenced their own. This included investigating differences between women who have and have not experienced abuse. Secondarily, we aimed to explore young women’s reported experiences of self-silencing and other partner-focused behaviors with their male partners, some of whom have committed abusive acts. Again, this involved examining differences based on IPV experience. Here, we hypothesized that IPV experience would be associated with higher self-silencing, partner demand/self-withdraw communication, and sexual compliance scores as well as lower constructive communication scores.

## Method

### Procedure

After receiving research ethics approval, we recruited young, heterosexual women aged 17 to 24 who studied and/or resided in Ontario, Canada, and who have been in an intimate relationship with a man for at least 6 months to participate in an online survey about experiences of conflict (and possibly experiences of abuse) in their intimate relationships. We employed multiple recruitment efforts, including paid advertising on Facebook and Instagram, posting in relevant Facebook groups, word-of-mouth, and after obtaining additional ethics approvals at various institutions, sending emails to students at select Ontario post-secondary institutions. The survey took about 15 to 20 minutes to complete, then participants were presented with a list of community resources and a document detailing browsing history deletion. Participants were also entered into a draw for a 1-in-5 chance of winning a $5 Starbucks e-gift card.

### Measures

In this study, we quantitatively measured young women’s experiences of abusive behaviors, self-silencing, communication, and sexual compliance, and qualitatively inquired about the way(s) that they silenced themselves in their intimate relationships. The Conflict in Adolescent Dating Relationships Inventory (CADRI; Wolfe et al., 2001) is a 35-item self-report measure of five types of abusive behaviors experienced among adolescent dating partners within the last year: verbal/emotional, threatening, relational, physical, and sexual. This measure was chosen for its focus on adolescent/youth audiences and included items that were relevant to young people’s relationships. A sample item from the threatening subscale is, “He deliberately tried to frighten me.” Participants responded on a 4-point Likert-type scale from 0 (*Never*) to 3 (*Often*). Possible scores ranged from 0-105, and scores were summed across abuse types. Internal consistency for the measure was .87 and test-retest reliability and construct validity have been established (e.g., Wolfe et al., 2001). In determining the presence of experienced IPV behaviors, we replicated the first author’s previous strategy for this categorization (Samardzic, 2019). For a participant to be included as having IPV experience, she needed to have answered with 1 (*Seldom*) or higher on at least one of any of the threatening, relational, physical, and/or sexual subscale questions *and/or* with 2 (*Sometimes*) or higher on at least one of the verbal/emotional subscale questions. Thus, the threshold was higher for verbal/emotional abuse.

The Silencing the Self Scale (STSS; Jack & Dill, 1992) is a 31-item self-report measure of self-silencing that came from Jack’s (1991) interviews with depressed women and purports to measure four discrete types of self-silencing behaviors: (a) Externalized Self-Perception (ESP), which involves self-judgment through external standards; (b) Care as Self-Sacrifice (CASS), which involves the over-prioritization of a partner’s needs over one’s own and active avoidance of being seen as selfish in the relationship; (c) Silencing the Self, which includes active inhibition of one’s self-expressions, such as voice; and the Divided Self (DS), an experience of having an inner angry self who is wishing to be heard and an outer, socially acceptable version of how a woman “should” be. A sample item from the CASS subscale is “Caring means putting the other person’s needs in front of my own.” Participants responded on a 5-point Likert-type scale from 1 (*Strongly Disagree*) to 5 (*Strongly Agree*), with higher scores indicating more self-silencing behaviors. Internal consistencies by subscale, in the order that they are described above, are as follows: α = .78, .55, .84, and 85, respectively; total score = .88). Test-retest reliability and construct validity have been established (e.g., Jack & Dill, 1992). Item-total correlations for items 1 and 11, which are CASS subscale items, in this study were .20 and .15, respectively. These values are higher than the correlations found previously by Samardzic (2019).

The Communication Patterns Questionnaire (Christensen, 1988; Christensen & Shenk, 1991; Christensen & Sullaway, 1984) is a 35-item self-report measure that assesses various communication styles and behaviors, both constructive and non-constructive, at three stages within the relationship: when a problem arises, while discussing a relationship problem, and after discussing a relationship problem. The measure includes three subscales: constructive communication, self-demand/partner withdrawal, and partner demand/self-withdrawal. Previous research suggests that both men (e.g., Pickover et al., 2017) and women (e.g., Uebelacker et al., 2003) engage in the demand/withdraw style, with partner demand/self-withdraw being associated with inflicting violence on their intimate partner and self-demand/partner withdraw involving an attempt to speak out in the face of violence. Given our research question and study focus, we focused on the constructive and partner demand/self-withdraw styles. A sample item from the latter is, “He threatens/you back down.” Participants answered on a 9-point Likert-type scale from 1 (*Very Unlikely*) to 9 (*Very Likely*). Internal consistency for the subscales was good (constructive: α = .82; partner demand/self-withdraw: α = .79) and psychometrics properties by subscale have been established (e.g., Heavey et al., 1996).

We assessed sexual compliance through three self-report items informed by previous works (e.g., Impett & Peplau, 2002). Participants were to reflect on the number of times that they engaged in undesired sexual activity without being pressured to do so. The items were as follows: (a) “How many times have you engaged in consensual sexual activity with your current partner because you felt like if you refused, the relationship would be damaged?”; (b) “How many other times have you engaged in sexual activity with your current partner even though you didn’t want to?”; and (c) “How many other times have you been in a situation with your current partner in which you consented to engage in sexual activity that you did not desire? In other words, your partner wanted to have sex, you did not want to, but you actually freely and willingly chose to do so anyway.” Participants answered based on the frequency of occurrence (0: 0 times; 1: 1–2 times; 2: 3–4 times; 3: 5–7 times; 4: 8+ times), with non-zero scores on one or more of the items being summed and indicating sexual compliance within their relationships.

We also had participants respond to five open-ended questions about behaving in ways that may be reflective of the silencing of their self-expression within the context of their current intimate relationship. These questions were developed by us and were informed by previous literature constructions of silence, care, and authenticity (e.g., Impett et al., 2008; Jack, 1991; Thompson et al., 2001). They are concerned with showing care (Q1), strategies for preventing conflict (Q2), disagreeing with one’s partner and not telling them (Q3), times where it was easier not to speak up (Q4), and being one’s true self in a relationship (Q5). Finally, participants provided demographic (i.e., racial/ethnic background, student status, living situation, how they heard about the study) and relationship (i.e., length of time with current partner and number of previous casual and committed intimate male partners) information.

### Participants

The sample consisted of 146 participants. Of those, 38 (26%) were determined to have not experienced any abusive behaviors (i.e., the No abuse [“NA”] group) and 108 (74%) had experienced at least one form of abuse (described above) at the hands of their intimate male partner (i.e., the Abuse [“A”] group). No significant differences in any demographic variables were observed among women who had and had not experienced abuse (see Table 1).

**Table 1.** Demographic and Relationship Information by Abusive Partner Behavior(s) Experienced.

Characteristic	Full “A” (*n* = 108), *M* (*SD*)	“NA” (*n* = 38*), *M* (*SD*)	Comparison between full “A” and “NA” groups^a^	Randomized “A” (*n* = 38), *M* (*SD*)	Comparison between randomized “A” and “NA” groups
Age	20.9 (2.0), *n* (%)	20.7 (1.9), *n* (%)	*t*(144) = −0.63, *p* = .53, 95% CI [−0.97, 0.50]	20.6 (1.8), *n* (%)	*t*(74) = 0.28, *p* = .78, 95% CI [−0.73, 0.97]
Racial/ethnic background					
White/European-Canadian	71 (66)	28 (74)	χ^2^ (8, *N* = 146) = 13.34, *p* = .10, ϕ = .30	26 (68)	χ^2^ (6, *N* = 76) = 9.07, *p* = .17, ϕ = .35
Middle Eastern	3 (3)	1 (3)		1 (3)	
South Asian	15 (14)	—		4 (11)	
Black	3 (3)	—		—	
Indigenous	4 (4)	—		2 (4)	
Latin or Central or South American	1 (0.5)	2 (5)		—	
West Asian	1 (0.5)	—		—	
East Asian	3 (3)	3 (7)		1 (3)	
Mixed race	7 (6)	4 (11)		4 (11)	
Student status					
Yes	105 (97)	37 (97)	χ^2^ (1, *N* = 146) = .002, *p* = .96, ϕ = −.004	38 (100)	χ^2^ (1, *N* = 76) = 1.01, *p* = .31, ϕ = .12
No	3 (3)	1 (3)		—	
Year of study					
High school	1 (1)	—	χ^2^ (6, *N* = 146) = 5.23, *p* = .52, ϕ = .19	2 (5)	χ^2^ (6, *N* = 76) = 5.03, *p* = .54, ϕ = .26
First-year	35 (33)	7 (19)		9 (24)	
Second-year	14 (13)	8 (22)		5 (13)	
Third-year	25 (24)	9 (24)		10 (26)	
Fourth-year	19 (18)	6 (16)		9 (24)	
Fifth-year	1 (1)	1 (2)		—	
Master’s	10 (10)	6 (16)		3 (8)	
Living situation					
With intimate partner	23 (21)	4 (11)	χ^2^ (5, *N* = 146) = 7.08, *p* = .22, ϕ = .22	5 (13)	χ^2^ (5, *N* = 76) = 7.50, *p* = .19, ϕ = .31
With roommates off-campus	31 (28)	19 (50)		9 (24)	
With roommates on campus	6 (6)	3 (8)		2 (4)	
With parent/relative/guardian(s)	43 (40)	11 (29)		20 (53)	
Alone	4 (4)	1 (2)		1 (3)	
Other	1 (1)			1 (3)	
Financial/work status					
Student	52 (48)	18 (47)	χ^2^ (5, *N* = 146) = 4.71, *p* = .45, ϕ = .18	16 (42)	χ^2^ (4, *N* = 76) = 1.93, *p* = .75, ϕ = .16
Employed part-time	42 (39)	13 (34)		15 (40)	
Employed full-time	6 (5)	1 (3)		3 (8)	
Unemployed, searching for work	4 (4)	2 (5)		2 (5)	
Unable to work	1 (1)	—		—	
Other	3 (3)	4 (11)		2 (5)	
Heard about study					
Post on Facebook	22 (20)	12 (32)	χ^2^ (3, *N* = 146) = 2.78, *p* = .43, ϕ = .14	9 (24)	χ^2^ (3, *N* = 76) = 2.56, *p* = .46, ϕ = .18
Post on Instagram	3 (3)	2 (5)		—	
From a friend or word-of-mouth	19 (18)	5 (13)		5 (13)	
Institutional email	64 (59)	19 (50)		24 (63)	
Relationship description					
Dating but not living together	81 (75)	30 (79)	χ^2^ (3, *N* = 146) = 1.19, *p* = .76, ϕ = .09	32 (84)	χ^2^ (3, *N* = 76) = 3.42, *p* = .33, ϕ = .21
Dating and living together	21 (20)	5 (13)		6 (16)	
Engaged	3 (2.5)	2 (5)		—	
Married or common-law	3 (2.5)	1 (3)		—	
Length of time with current partner^b^	29.3 (20.4)	27.2 (20.9)	*t*(144) = −0.55, *p* = .58, 95% CI [−9.79, 5.52]	26.5 (14.7)	*t*(74) = 0.17, *p* = .86, 95% CI [−7.54, 8.96]
Engagement in sexual activity with a man					
Yes	104 (96)	35 (92)	χ^2^ (1, *N* = 146) = 1.08, *p* = .30, ϕ = .09	38 (100)	χ^2^ (1, *N* = 76) = 3.12, *p* = .08, ϕ = .20
No	4 (4)	3 (8)		—	
Number of committed relationships	1.8 (1.0)	1.6 (0.8)	*t*(143) = −1.29, *p* = .20, 95% CI [−0.57, 0.12]	1.8 (0.9)	*t*(74) = −1.11, *p* = .27, 95% CI [−0.59, 0.17]
Number of casual relationships	2.7 (4.0)	1.8 (2.4)	*t*(143) = −1.28, *p* = .20, 95% CI [−2.24, 0.48]	2.1 (2.2)	*t*(74) = −0.75, *p* = .46, 95% CI [−1.45, 0.66]

*Note.* “A” is the abuse-experienced group while “NA” is the no abuse-experienced group.

* Three missing from the “NA” group.

a The statistic for the comparison was either a *t*-test or chi-square test depending on the type of variable. To reduce the chances of type I error, we employed a Bonferroni correction for the chi-square tests (new *p* = .01 for each of the full “A” vs. “NA” and randomized “A” vs. “NA” groups) for the demographic information. We did two other Bonferroni corrections for the *t*-tests (new *p* = .004 for both sets of comparisons, some of which are reported in Table 3) and chi-square tests (new *p* = .03 for both sets of comparisons) for the relationship information.

b Length of time in months. In years, the averages would be 2.5 years (*SD* = 1.7) for the full “A” group, 2.3 years (*SD* = 1.7) for the “NA” group, and 2.2 years (*SD* = 1.3) for the randomized “A” group.

### Data Analysis

#### Primary Study Aim: Content Analysis

To analyze and compare the content within participants’ descriptions of situations where they engaged in over-prioritizing their partner and his needs at their own expense, we conducted a content analysis. Because we had an unequal number of women in both groups, we randomly selected 38 young women from the “A” group using a random item generator website (https://miniwebtool.com/random-picker/) and listed the participant numbers of the women in the “A” group. Every time a participant number was randomly selected, we removed them from the remaining list and re-selected a random participant, repeating the process until we had 38 young women in each group. The words per open-ended question ranged from 1 to 372, with the average words by question ranging from 47 to 83 and the average number of words collated across questions was 63.1 (*SD* = 51.2). No significant between-group differences in the number of words by open-ended question or any demographic or relationship variables existed.

We completed a mixed inductive and deductive content analysis on young women’s responses to the open-ended questions about care, disagreement, speaking up versus not speaking up, and authenticity in their intimate relationships (Hsieh & Shannon, 2005). The codes were informed from the extant literature (e.g., Impett et al., 2008; Jack, 1991) and participants’ responses. The first author created the first draft of a coding rubric, on which the second author provided feedback. The number of codes by question varied: Q1: 8, Q2/3: 10 each, Q4: 9, and Q5: 7. Codes were identified based on knowledge of the silencing literature (e.g., prioritizing, self-suppressing, feeling angry when not being able to be authentic; Jack, 1991) and commonalities in the other “A” group data that were not included in the analyses. Coders were instructed that they could code the same unit of data within multiple codes (e.g., in situations where someone said that they are communicative but have suppressed parts of themselves in the past). In situations where a unit of data did not fit any of the coding categories (e.g., contextual details that are part of a participant’s description of an event), coders were instructed that they should not code those. Finally, if a participant said “see previous answer” or something similar, coders were instructed to leave that blank because it would have already been coded once.

The updated coding rubric was then brought to the coding team, who described themselves as a 20-year-old first-generation South Asian Canadian and a 22-year-old White, queer Canadian student. Coders conducted two practice rounds on unused data from the “A” group and made-up data. Through the practice rounds, some of the coding descriptions were refined to include more specificity given the confusion among the coders. They practiced until the percentage agreement for all variables exceeded 70% and each reported comfort with the rubric before conducting the coding presented here.

To assess inter-rater reliability, we calculated percentage agreement and Gwet’s Agreement Coefficient (AC1). The latter was chosen over Cohen’s kappa given the volatility of kappa when there is a high percentage of agreement between reviewers (e.g., Gwet, 2008). This paradoxical situation misrepresents the data, so Gwet’s AC1 was the preferred statistic here and elsewhere (e.g., Collaton et al., 2022). Percentage agreement and Gwet’s AC1 were calculated in RStudio v.4.2.1 (PBC, Boston, Massachusetts, United States) using the “irrCAC” package and ranged from 79% to 100% across codes (93% agreement total across all questions and codes). Overall, most of the inter-rater reliability scores were very good, one was good, and one was moderate (AC1 = 0.89, 95% CI [0.88, 0.91]) across all questions and variables (see Table 2).^2^ The first author’s original coding of the data was used to record the final tally. This was after all instances of disagreement were reviewed to ensure that each data point did belong in its original category.

**Table 2.** Content Analysis by Open-Ended Question and Abusive Partner Behavior(s) Experienced.

Codes	“A” (*n* = 38), *n* (%)	“NA” (*n* = 38), *n* (%)	Statistic^a^	% Agreement	Gwet’s AC1 [95% CI]^b^
Question (Q)1: Showing care				95%	0.91 [0.87, 0.94]
Acts of service: *doing things that service or help their partner in some way (e.g., running an errand for him)*	15 (40)	11 (29)	χ^2^ (1, *N* = 76) = .51, *p* = .47, ϕ = .08	94%	0.90 [0.79, 1.00]
Words of affirmation: *ways of communicating their feelings (e.g., “I love you”)*	12 (32)	12 (32)	χ^2^ (1, *N* = 76) = .06, *p* = .81, ϕ = −.03	97%	0.95 [0.88, 1.00]
Physical touch: *(non)sexual touches (e.g., kisses, massages)*	15 (40)	11 (29)	χ^2^ (1, *N* = 76) = .51, *p* = .47, ϕ = .08	96%	0.92 [0.83, 1.00]
Gift-giving: *purchasing something for their partner (e.g., a coffee)*	11 (29)	9 (24)	χ^2^ (1, *N* = 76) = 1.00, *p* = .76, ϕ = .04	96%	0.93 [0.83, 1.00]
Checking in/communication: *checking in to see how their partner was doing, including communication via phone (e.g., texting)*	16 (42)	22 (58)	χ^2^ (1, *N* = 76) = 3.14, *p* = .08, ϕ = −.21	90%	0.81 [0.67, 0.95]
Quality time: *going on dates and enjoying communal activities*	18 (47)	14 (37)	χ^2^ (1, *N* = 76) = .40, *p* = .53, ϕ = .07	96%	0.92 [0.82, 1.00]
Support: *participants supporting their partner (e.g., giving advice and supporting his mental health)*	13 (34)	16 (42)	χ^2^ (1, *N* = 76) = 1.01, *p* = .32, ϕ = −.12	92%	0.85 [0.72, 0.97]
*Prioritizing him: *putting their partner’s needs ahead of their own, particularly at their own expense (e.g., changing their own plans to ensure that their partner’s plans are intact)*	3 (8)	1 (3)	χ^2^ (1, *N* = 76) = .89, *p* = .35, ϕ = .11	97%	0.97 [0.92, 1.00]
Q2: Strategies to prevent conflict				94%	0.92 [0.90, 0.95]
*Checking on his feelings: *participants focus on how their partners feel in the conflict*	5 (13)	4 (11)	χ^2^ (1, *N* = 76) = .05, *p* = .82, ϕ = .03	94%	0.94 [0.87, 1.00]
Taking space/a breather: *one or both of them taking a moment to step away from the conflict and compose themselves*	6 (16)	7 (18)	χ^2^ (1, *N* = 76) = .22, *p* = .64, ϕ = −.06	92%	0.88 [0.78, 0.98]
Apologizing: *a participant apologizing to their partner during a conflict*	2 (5)	1 (3)	χ^2^ (1, *N* = 76) = .27, *p* = .61, ϕ = .06	100%	1.00
Changing the subject: *a participant changing the subject during a conflict with their partner*	1 (3)	1 (3)	χ^2^ (1, *N* = 76) = .003, *p* = .95, ϕ = −.01	100%	1.00
Communication about issues: *both parties willingly and comfortably discussing issues together*	16 (42)	21 (55)	χ^2^ (1, *N* = 76) = 2.33, *p* = .13, ϕ = −.18	85%	0.70 [0.53, 0.87]
*Suppressing some part(s) of oneself: *keeping some part(s) of themselves hidden, secret, or quiet (e.g., saying “I’m fine” instead of telling their partner what is going on)*	20 (53)	9 (24)	χ^2^ (1, *N* = 76) = 5.51, *p* = .02, ϕ = .28	94%	0.89 [0.79, 1.00]
*Mental work: *internal thought processes concerned with thinking through the implications of bringing something up to their partner (e.g., will he think I am jealous or crazy if I tell him?)*	8 (21)	8 (21)	χ^2^ (1, *N* = 76) = .04, *p* = .85, ϕ = −.02	92%	0.89 [0.79, 0.98]
*Onus on oneself: *tangible strategies that participants undertook to take responsibility for the conflict (e.g., discussing what she could do better going forward)*	3 (8)	3 (8)	χ^2^ (1, *N* = 76) = .01, *p* = .92, ϕ = −.01	86%	0.81 [0.69, 0.94]
Putting off conflict until an appropriate time: *deliberately putting off conflict because it is not the right time to discuss things (e.g., they are out with friends or family)*	—	1 (3)	χ^2^ (1, *N* = 76) = 1.10, *p* = .29, ϕ = −.12	100%	1.00
Getting a third party’s perspective: *engaging someone else (e.g., a friend or therapist) and discussing their conflict with them*	1 (3)	1 (3)	χ^2^ (1, *N* = 76) = .003, *p* = .95, ϕ = −.01	99%	0.99 [0.96, 1.00]
Q3: Disagreeing and keeping quiet				**92%**	**0.88 [0.85, 0.92]**
*Fear: *expression of anxiety, nervousness, fear, worry, etc. about their partner’s feelings (e.g., worry about stressing him out before an exam if they bring something up) and/or the integrity of the relationship (e.g., worry that he will get angry if they bring something up)*	6 (16)	8 (21)	χ^2^ (1, *N* = 76) = .59, *p* = .44, ϕ = −.09	94%	0.91 [0.83, 1.00]
*Frustration: *participants experiencing anger, annoyance, resentfulness, etc.*	6 (16)	9 (24)	χ^2^ (1, *N* = 76) = .48, *p* = .49, ϕ = .08	99%	0.98 [0.94, 1.00]
*Upset: *participants being sad, engaging in crying, etc.*	9 (24)	15 (39)	χ^2^ (1, *N* = 76) = 1.56, *p* = .21, ϕ = .15	92%	0.86 [0.74, 0.97]
*Prioritizing his needs: *things like participants knowing something means a lot to him and not wanting to ruin it, trying to see things from his side, etc.*	4 (11)	5 (13)	χ^2^ (1, *N* = 76) = .29, *p* = .59, ϕ = −.06	92%	0.90 [0.82, 0.99]
Communication about issues: *same as Q2*	15 (39)	16 (42)	χ^2^ (1, *N* = 76) = 29, *p* = .59, ϕ = −.06	89%	0.79 [0.64, 0.93]
*Not speaking up to avoid disagreement: *not wanting to speak up to avoid causing a fight or blowing things out of proportion*	6 (16)	8 (21)	χ^2^ (1, *N* = 76) = .59, *p* = .44, ϕ = −.09	79%	0.65 [0.47, 0.83]
*Not speaking up to avoid his reaction: *his negative reaction (e.g., anger) dissuading them from speaking up*	5 (13)	3 (8)	χ^2^ (1, *N* = 76) = .39, *p* = .53, ϕ = .07	93%	0.92 [0.84, 0.99]
*Mental work: *same as Q2*	6 (16)	6 (16)	χ^2^ (1, *N* = 76) = .02, *p* = .88, ϕ = −.02	83%	0.79 [0.656, 0.92]
Putting off conflict until an appropriate time: *same as Q2*	—	3 (8)	χ^2^ (1, *N* = 76) = 3.40, *p* = .07, ϕ = −.22	97%	0.97 [0.93, 1.00]
Not speaking up led to more conflict: *instances where conflict escalated because a participant did not speak up and recognized that speaking up would have mitigated the conflict altogether*	—	3 (8)	χ^2^ (1, *N* = 76) = 3.40, *p* = .07, ϕ = −.22	100%	1.00
Q4: Easier to not speak up				**91%**	**0.88 [0.84, 0.91]**
Communication about issues: *same as in Q2 and Q3*	9 (24)	10 (26)	χ^2^ (1, *N* = 76) = .23, *p* = .63, ϕ = −.06	95%	0.91 [0.814, 1.00]
*Not speaking up to avoid making him and/or others feel bad: *a participant not wanting to speak up for reasons like possibly ruining an event for their partner and his friends or not wanting to hurt their partner’s feelings*	6 (16)	5 (13)	χ^2^ (1, *N* = 76) = .03, *p* = .86, ϕ = .02	92%	0.89 [0.79, 0.98]
*Not speaking up to avoid disagreement: *same as in Q3*	9 (24)	4 (11)	χ^2^ (1, *N* = 76) = 1.87, *p* = .17, ϕ = .16	90%	0.86 [0.76, 0.97]
*Not speaking up to avoid his reaction: *same as in Q3*	8 (21)	7 (18)	χ^2^ (1, *N* = 76) = .01, *p* = .91, ϕ = .01	89%	0.85 [0.74, 0.96]
*Suppressing things that are not worth bringing up: a *participant deciding to not bring something up because there is no point (e.g., a previously discussed topic that never gets resolved)*	7 (18)	12 (32)	χ^2^ (1, *N* = 76) = 2.38, *p* = .12, ϕ = −.18	88%	0.79 [0.65, 0.93]
*Mental work: *same as in Q2 and Q3*	6 (16)	2 (5)	χ^2^ (1, *N* = 76) = 1.90, *p* = .17, ϕ = .16	89%	0.87 [0.78, 0.97]
*Onus on oneself: *same as in Q2*	2 (5)	2 (5)	χ^2^ (1, *N* = 76) = .01, *p* = .93, ϕ = −.01	88%	0.84 [0.73, 0.95]
Partner dismissive of her feelings: *a participant communicating their feelings and/or him knowing the participant is upset and him denying or minimizing his role, demeaning her, etc.*	9 (24)	4 (11)	χ^2^ (1, *N* = 76) = 1.87, *p* = .17, ϕ = .16	97%	0.96 [0.91, 1.00]
N/A: *the question did not apply to the participant and their relationship*	7 (18)	9 (24)	χ^2^(1, *N* = 76) = .57, *p* = .45, ϕ = −.09	94%	0.92 [0.83, 1.00]
Q5: Being their true, authentic self				93%	0.89 [0.84, 0.92]^c^
*Ability to be one’s true self: *being able to be their true self in their current relationship*	28 (74)	32 (84)	χ^2^ (1, *N* = 76) = 3.92, *p* = .05, ϕ = −.23	97%	0.96 [0.90, 1.00]
*Not being able to be one’s true self: *identifying that a participant cannot be their true self either some or all of the time*	10 (26)	3 (8)	χ^2^ (1, *N* = 76) = 3.03, *p* = .08, ϕ = .20	97%	0.96 [0.90, 1.00]
*Being able to be one’s true self with him, unlike others: *discussion of a participant’s inability to be their true self with an ex-partner but being able to be their true self in the current relationship*	5 (13)	5 (13)	χ^2^ (1, *N* = 76) = .02, *p* = .89, ϕ = −.02	92%	0.89 [0.80, 0.98]
Encouragement/support from their partner: *their partner being supportive, accepting, etc. in the participant being their true self*	16 (42)	25 (66)	χ^2^ (1, *N* = 76) = 6.36, *p* = .01, ϕ = −.30	82%	0.56 [0.36, 0.75]^d^
*Personal factors hindering them: *factors (e.g., mental health concerns, impacts of her previous relationship) that impact the participant’ ability to be their true self in the current relationship*	6 (16)	3 (8)	χ^2^ (1, *N* = 76) = .88, *p* = .35, ϕ = .11	97%	0.96 [0.91, 1.00]
*Partner impacts ability to be one’s true self: *times when a participant’s partner has made it difficult for them to be their true self (e.g., times when he was judgmental or reacted angrily)*	2 (5)	1 (3)	χ^2^ (1, *N* = 76) = .27, *p* = .61, ϕ = .06	97%	0.97 [0.93, 1.00]

*Note:* Three missing cases. Significant differences are bolded.

* Refers to codes that reflect silencing-type behaviors. “A” is the abuse-experienced group and “NA” is the no abuse-experienced group.

a The effect size of choice for chi-square tests was a phi-coefficient, which estimates the degree of association between the rows and columns. Phi-coefficients range from −1.00 to 1.00 and operate much like correlation coefficients.

b Gwet’s AC1 value is considered “very good” if it is within the 0.80 to 1.00 range, “good” if it is in the 0.60 to 0.80 range, and “moderate” if it is in the 0.40 to 0.60 range (Gwet, 2014).

c “No hiding” (i.e., discussions of them not needing to hide any part of themselves from their partner) was removed from Q5 given its low score (0.46) and percentage agreement (71%). The updated Gwet’s AC1 for Q5 is displayed in this table.

d It may be that the level of agreement on this code was lower due to raters not differentiating between instances where young women reported that their partner was encouraging to and supportive of them being their true selves and/or doing things in life that make them happy and instances where young women referenced that both parties work together to ensure openness and communication. The latter situation was sometimes coded here, which likely affected the agreement.

#### Exploratory Analyses

We also aimed to explore young women’s experiences of self-silencing and other partner-focused behaviors both overall and compared by presence versus absence of abuse experience. We used IMB SPSS v.28 (Armonk, New York, United States) to run descriptive statistics on the main study variables and difference tests on sociodemographic, relationship, and main study variables between the groups of women who had and had not experienced IPV. We conducted chi-square tests for categorical variables (e.g., school status) and independent samples *t*-tests for continuous variables (e.g., age).

## Results

### Primary Study Aim: Content Analysis

Our primary aim was to analyze the content present in young women’s descriptions of their intimate relationships with their male partners. We compared written descriptions by IPV experience with the matched sample sizes. While many of the codes present in young women’s open-ended responses were not specific to silencing as described in the literature, our analysis focuses on those relevant to them silencing themselves (asterisked in Table 2).

#### Showing Care

Of the eight codes pertinent to this question, only one concerned women silencing themselves by prioritizing their partners. However, only 3% of the “NA” and 8% of the “A” samples reported behaving as such. More commonly, both described behaviors like checking in/communicating with their partner (“NA”: 58%; “A”: 42%), spending quality time with (“NA”: 37%; “A”: 47%) and supporting their partner (“NA”: 42%; “A”: 34%).

#### Strategies to Prevent Conflict

Of the 10 codes for this question, only four were examples of women silencing themselves. Overall, there were no notable differences between the two groups on most behaviors. That said, some women, in addition to self-suppression, also engaged in mental work or took the onus on themselves.

#### Disagreeing and Keeping Quiet

Seven of these 10 codes for this question reflected women silencing themselves. Women in the “NA” group felt more fearful (21% vs. 16%), upset (39% vs. 24%), and frustrated (24% vs. 16%) compared to women in the “A” group. Also, the reason for women in the “NA” group choosing to silence was more often to avoid causing a disagreement (21% vs. 16%). Slightly more women in the “A” (13%) compared to the “NA” group (8%) reported not wanting to speak up to avoid their partner’s reaction. Generally, the endorsement of these codes was low, with many more women in both groups indicating that they instead communicate with their partner (“NA”: 42%; “A”: 39%).

#### Easier to Not Speak Up

Of the nine codes for this question, six were examples of times where women silenced themselves. Here, more participants in the “A” group engaged in mental work (16% vs. 5%), and as such, those participants more often indicated that their partner was dismissive of their feelings (24% vs. 11%). While such silencing-type behaviors were more common in the “A” group for this question, again, the extent to which women reported behaving in these ways was low regardless of group (i.e., usually ≤25% of total participants).

#### Being One’s True, Authentic Self

Most participants in the “NA” (84%) and “A” (74%) groups reported being able to be their authentic selves in their relationships. Fewer women specified that they could not be their true selves and there was a noteworthy but non-significant difference between groups with more women in the “A” group (26% vs. 8%) indicating that they could not be their true selves. For those who indicated not speaking up, it was slightly more common to not do so to avoid a disagreement (32%) than their partner’s reaction (30%).

### Exploratory Analyses

We also explored the quantitative data for the commonality of self-directed behaviors (e.g., silencing-type behaviors and compliance with unwanted sex) and experience of partners’ behaviors (i.e., harmful and possibly abusive behaviors) from the full online sample (“NA”: *n* = 38; “A”: *n* = 108). Participants’ averaged total STSS score was 75.6 (range: 46–140), which falls in the middle of the STSS ranges from possible scores of 31 to 155. Among the facets of self-silencing, the highest score was CASS (*M* = 23.7, range: 13–36), and the lowest was the DS (*M* = 12.2, range: 7–32). We also explored differences between our two groups across the outcome measures. As hypothesized, participants in the “A” group reported higher total self-silencing, DS, partner demand/self-withdraw communication, and sexual compliance compared to the “NA” group, and these differences were statistically significant. As hypothesized, they also reported significantly less constructive communication than “NA” group members (see Table 3).

**Table 3.** Exploratory Analyses by Abusive Partner Behavior(s) Experienced.

Outcome	Full “A” (*n* = 108), *M* (*SD*)	“NA” (*n* = 38*), *M* (*SD*)	Comparison (*t*-test)^a^
Conflict in adolescent dating relationships inventory (frequency)	10.9 (9.2)	1.3 (1.2)	****t**(144) = −6.39, *p* < .001, 95% CI [−12.55, −6.62]**
Verbal/emotional	93 (86)	—	
Threatening behavior	17 (16)	—	
Relational	14 (13)	—	
Physical	18 (17)	—	
Sexual	49 (45)	—	
Silencing the self scale (frequency)			
Externalized self-perception	21.0 (5.1)	18.9 (4.6)	*t*(144) = −2.10, *p* = .02, 95% CI [−3.81, −0.11]
Care as self-sacrifice	23.7 (5.0)	23.6 (4.1)	*t*(144) = −.09, *p* = .47, 95% CI [−1.85, 1.69]
Silencing the self	21.3 (7.6)	18.5 (5.7)	*t*(144) = −2.06, *p* = .02, 95% CI [−5.45, −0.11]
Divided self	14.9 (6.4)	9.5 (2.6)	***t*(144) = −5.05, *p <* .001, 95% CI [−7.49, −3.28]**
Total score	80.7 (17.6)	70.1 (11.7)	***t*(144) = −3.31, *p <* .001, 95% CI [−16.25, −4.10]**
Sexual compliance (frequency)	4.1 (3.6)	2.0 (2.9)	*t*(144) = −3.39, *p* < .001, 95% CI [−3.44, −0.91]
Communication patterns questionnaire (frequency)			
Constructive	60.0 (12.2)	68.1 (7.4)	***t*(144) = 3.86, *p <* .001, 95% CI [3.94, 12.25]**
Partner demand/self-withdraw	22.2 (9.2)	13.3 (6.1)	***t*(144) = −5.56, *p <* .001, 95% CI [−12.04, −5.72]**

*Note*. “A” is the abuse-experienced group while “NA” is the no abuse-experienced group. Little’s (1988) MCAR test indicated that the data were missing completely at random: χ2 (17, *N* = 146) = 17.36, p = .43. We used expectation maximization, which generates imputed values that are consistent with population values (Shafer, 1997), to replace the missing values ahead of the exploratory analyses. Significant differences are bolded.

* Three missing from the “NA” group.

a To reduce the chances of type I error, we employed a Bonferroni correction (new *p* = .004).

## Discussion

Our study aims were (a) examination of the content within young women’s descriptions of the situations where they over-prioritized their partner’s needs at the expense of their own within their intimate relationships, and (b) an exploratory comparison of young women’s reported experiences of self-silencing and other partner-focused behaviors within relationships where abuse had and had not been experienced. In their qualitative responses, several young women emphasized communication as integral to relationships, and, regardless of IPV experience, most reported feeling as though they could be their authentic selves with their partners. This complicates the seemingly straightforward exploratory quantitative findings, which showed that young women who had experienced IPV reported higher partner-focused behaviors of every type that was measured. These women also reported less constructive communication in their relationship, which supported our hypothesis.

The content within our participants’ open-ended responses suggests two important things. First, they seemed to regard communication as necessary and helpful, even when it might increase conflict, as evidenced by their overwhelming endorsement of communication in conflict situations regardless of IPV experience (e.g., use of “always” when referencing communication and noting the impact of communication for their relationship). It was also common for them to suppress themselves to reduce or avoid conflict. This differs from other studies, such as Cormier’s (2004) interviews, where participants highlighted a devaluation of their experiences, voice, and feelings and became disconnected from their sense of self. It is common in the self-silencing literature to see descriptive language like women experiencing a “loss of voice” (Jack, 1991). Our participants’ responses, however, suggested that they intentionally silenced themselves in specific instances, rather than had an all-encompassing experience of silencing themselves constantly. Still, communication was the most common strategy regarding decisions to speak up and not speak up as well as conflict management and resolution, which has been seen elsewhere (e.g., Debman et al.’s [2014] interviews with African American girls). This strong endorsement of communication may also reflect a shift in social constructions of what is desirable in all relationships (e.g., work and dating), thus casting silence and inauthenticity in a negative light and contributing to possible under-reporting (e.g., Erickson, 2021; Samardzic et al., 2023). Further research exploring the possibility of social desirability when responding to questions such as ours would help further contextualize the strong endorsement of communication.

Second, most of the young women whose responses were included in the content analysis indicated that they felt as though they could be their true, authentic selves in their intimate relationships. Impett et al. (2008) conceptualized relationship authenticity as a congruence between a young woman’s thoughts and feelings and what she says and does in a relational context. Several participants’ responses reflected an incongruence despite their assertions that they felt they could be their true selves. This aligns with their quantitative scores given that the most highly endorsed question across the sample was “My partner loves and appreciates me for who I am” (*M* = 4.7/5). Interestingly, of the 60 women who indicated experiencing such authenticity, one-third reported self-suppressing (Q2), 38% engaged in mental work (Q3/4), 28% indicated suppressing things they deemed not being worth speaking up about, and almost half described not speaking up to avoid a disagreement and/or a negative reaction (Q3/4). But their average item scores for both the abuse and non-abuse groups scores (avg. = 2.5/5) are incongruent. It may be that their assessment was more global of the entire relationship whereas the open-ended responses were situation-specific, and their silencing may therefore be sporadic and situational. Thus, our findings make us question whether anyone can be their true self in a relationship at all times. The answer is that they likely cannot, for reasons such as the existing contradictions between people’s thoughts and actions (e.g., Gavey, 1989). Future researchers may wish to explore the nature of young women’s sense of authenticity more globally as well as employ methods that afford an understanding of the complexities inherent in people’s views and experiences within the world (e.g., a feminist post-structural discourse analysis; Gavey, 1989).

Self-silencing and related behaviors were common, especially among those with IPV experience, as demonstrated by our exploratory quantitative analyses. However, when compared to previous literature, our participants’ self-silencing scores were lower. This is observed in participants’ responses to the STSS questions that inquired about speaking up versus not speaking up, which were often not as highly endorsed (*M* = 2.3/5). Compared with previous research studies on undergraduate students who had not experienced IPV, like Gratch et al.’s (1995) sample of college students from the southwestern United States who had a mean age of 24 (STSS total *M* for women in their sample = 77.5) and Hurst and Beesley’s (2013) sample of undergraduates aged 18 to 29 (STSS total *M* = 82.2), our total STSS score was lower (STSS total *M* for the “NA” group = 70.1). Fewer studies reported subscale scores, though when comparing with Duarte and Thompson’s (1999) study with Canadian undergraduates with an average age of 22, there were generally similar scores except for our participants’ higher CASS scores (ours: 23.6; their women’s sample: 18.4) and lower DS scores (ours: 9.5; their women’s sample: 15.0).

It was much more difficult to find an appropriate comparison for our sample of young women who had experienced IPV as these other studies tended to include much older women (e.g., average age at least in their mid-30s) who often met clinical criteria for various diagnoses, like depression or bulimia, and/or were involved with IPV supports in the community. But looking at a few studies, like Woods’ (1999) work with women survivors of abuse who had an average age of 32 and Neves and Nogueria’s (2010) work with Portuguese women in the 35 to 45-year age group, our “A” group scores tend to be lower. For instance, Woods’ (1999, 2012) total STSS score was 105.5, Neves and Nogueria’s (2010) total score was 86.4, and our “A” group total score was 80.7. Especially notable was our lower DS score (*M* = 14.9) compared to Woods’ (*M* = 27.4). Indeed, the most significant finding from our exploratory analyses was our participants’ DS scores being much lower than what had previously been measured quantitatively, regardless of IPV experience. Relatedly, the extent of our participants’ discussion of an experienced division of self was low compared to previous qualitative work (e.g., Jack & Ali, 2010). This is especially interesting when coupled with the findings that women reported lower sexual compliance and partner demand/self-withdraw communication scores and generally higher constructive communication scores compared to past research (e.g., Pickover et al., 2021).

Taken together, these findings suggest that young women today may feel more empowered to speak out against relational injustices compared to similar samples of young, predominantly White, heterosexual women recruited from post-secondary institutions and other areas of the community in the 1980s to early 2000s when much of this research occurred. However, there were likely important differences between those samples and our own, including generational differences, clinical samples, and how the studies were conducted, to name a few. For example, many of our participants were highly educated, which may have afforded them a deeper understanding of the relational injustices that they were experiencing in their relationships, as well as the language to identify those injustices. This may not have been the case in other studies that were conducted decades ago. Leveraging mixed methods to explore the commonality of these behaviors and also having qualitative data to provide additional context to their quantitative responses was important to nuance and further unpack experiences of women silencing themselves in intimate relationships where abuse had and had not occurred.

### Strengths and Limitations

Our study includes some limitations. First, the abuse sample is a product of our recruitment efforts and categorization choices. We used both euphemistic and direct language (e.g., “stressful experiences in their intimate relationships . . . including possible experiences of abuse”) when recruiting. It is therefore unsurprising that the sample mostly comprised young women with one or more forms of IPV experienced, including a need to have had more than one instance of emotional abuse on the adolescent abuse experience scale that we used (i.e., the most commonly experienced form of abuse in this study, despite there being a higher threshold to reach) to qualify. This was itself a strength given that women without IPV experiences still responded to an advertisement about conflict and we could compare what conflict experiences looked like in both groups. Future researchers may wish to first employ two recruitment methods, one with euphemistic language and one that asks about relationship experiences more generally, to get a more evenly matched sample of women with and without IPV experience. We also used a measure that was not intended to be used for categorization purposes. A strength of this, which was developed in consultation with other literature (e.g., Marshall, 1992), was that it allowed us to include young women who had experienced abusive acts but who may not label their partners as abusive. Researchers may also wish to use an IPV measure with specific categorization instructions, such as the Composite Abuse Scale (Hegarty et al., 1999), which is a multidimensional IPV measure with four subscales. This measure would allow ratings of the amount and frequency of IPV within the past year, resulting in more response variance. Nevertheless, the CADRI was used because it was specifically developed to be used with youth.

Our study also lacked diversity, especially regarding participants’ racial/ethnic backgrounds (68% White) and student status (97% students). Despite the substantial effort to recruit a more diverse sample, we received little response and thus focused on recruiting from post-secondary institutions given our age range of interest. A more diverse sample could provide nuance to topics of conflict management and decisions regarding speaking up. Also, relationship context diversity (e.g., living situations) could provide future insights, especially since women who live with their abusers are at higher risk of experiencing IPV (Renner & Whitney, 2012). To increase diversity, employing strategies like using more personalized recruitment advertisements (González & Grov, 2022) and connecting with organizations that work with diverse young women in the community to have them aid in the recruitment process may be necessary.

### Directions for Future Research

Our findings both illuminate the continued prevalence of silence and underscore the importance of further exploring the phenomenon, specifically in the context of women with lived experience of IPV. In this study, we saw differences in women’s silencing of themselves between the two groups and the manifestation of that silencing was that it may not be as all-encompassing and thus as damaging to a relationship as it has previously been conceptualized (e.g., Brown & Gilligan, 1992; Jack, 1991). Gilligan (2018) suggested that important social movements such as #MeToo have allowed women to break their silence and therefore use their voices more publicly. However, amid the continual societal expectations concerning how women “should” be in their intimate, heterosexual relationships, little is known about the decision-making process and the consequences of speaking up versus not speaking up. What this study could not illuminate is under what circumstances women with IPV experience choose to speak out versus not as well as other questions about silencing oneself, such as whether it is the context in which it occurs (e.g., the IPV context) that impacts and perhaps is damaging to a relationship. It is also unclear what creates the conditions for them to speak out to their abusive male partner. As such, employing a methodological approach such as reflexive thematic analysis (Braun & Clarke, 2019) would allow an understanding of the ways that women experience silencing themselves and speaking out from their point of view and in their unique context. Unpacking the experiences of young women who have faced IPV in a way that provides more detail than was afforded by our open-ended questions (e.g., interviews) could extend findings from other studies on young women who are in heterosexual relationships where their partners are abusive.

## Conclusion

This work provides an updated account of the continued impact and importance of silencing the self in young, heterosexual women, something that has recently been demonstrated (e.g., Avery et al., 2022). Our quantitative findings show a clear relationship between self-silencing and IPV, but this is complicated somewhat by our examination of the context in the qualitative content analysis. Here, despite communication being emphasized as a favored way to manage conflict and maintain their relationship, women silenced themselves in different ways. Our findings suggest that silencing oneself may be more situational compared to previous framings of it being all-encompassing and suggestive of detrimental impacts to the relationship, such as an inability to be one’s true self, which most of our study participants felt they could be. We offer future research suggestions, namely further exploring women silencing themselves in the context of IPV to understand the conditions under which they choose to speak out or not in their relationships.

## Notes

1. In this manuscript, it was important for us to differentiate between “self-silencing” as the specific construct that has been coined, conceptualized, and measured quantitatively in the psychological literature (Jack & Dill, 1992) and women silencing themselves as engagement in silencing behaviors, and/or some variation of silencing to refer to the restriction of self-expressions for the goal of relationship maintenance outside of the boundaries placed by Jack and Dill. We also wanted to avoid nominalizing self-silencing as if it exists in real life as a quantifiable psychological variable, rather than a set of ways in which women negotiate their relationships in an agentic way. We achieved this by phrasing women’s behavior in a way that reflects their agency and/or the socially contingent ways in which they act.

2. “No hiding” was removed from Q5 given its low score (0.46 [0.25, 0.68]) and percentage agreement (71%). The previous total AC1 score with “no hiding” was 0.82 (0.78, 0.87).
